# Editorial: composite materials in biological and bioinspired systems

**DOI:** 10.1098/rsfs.2024.0008

**Published:** 2024-04-12

**Authors:** Wencke Krings, Stanislav N. Gorb

**Affiliations:** ^1^ Department of Cariology, Endodontology and Periodontology, Universität Leipzig, Liebigstraße 12, 04103 Leipzig, Germany; ^2^ Department of Electron Microscopy, Institute of Cell and Systems Biology of Animals, Universität Hamburg, Martin-Luther-King-Platz 3, 20146 Hamburg, Germany; ^3^ Department of Mammalogy and Palaeoanthropology, Leibniz Institute for the Analysis of Biodiversity Change, Martin-Luther-King-Platz 3, 20146 Hamburg, Germany; ^4^ Department of Functional Morphology and Biomechanics, Zoological Institute, Christian-Albrechts-Universität zu Kiel, Am Botanischen Garten 1–9, 24118 Kiel, Germany

**Keywords:** composite, materials, biological, bioinspired

The research field devoted to biological composite materials represents an intersection between biology, materials science and engineering. These materials are composed of two or more components with distinct properties, which in combination lead to enhanced or novel properties meeting specific mechanical, physiological or ecological needs of organisms. In recent years, there has been growing interest in the use of biologically inspired composite materials in fields such as biomedical engineering, aerospace, automotive and sustainable construction. In the biomedical area, these materials are being used as tissue engineering scaffolds, drug delivery systems and implantable medical devices. By mimicking the structure and composition of biological materials, researchers aim at development of biocompatible materials that can be seamlessly integrated with the body, promoting tissue regeneration and healing. In aerospace and automotive industries, bioinspired composite materials offer new avenues for lightweight, high-strength alternatives to traditional composites. By direct incorporation of biological materials, such as cellulose, lignin or chitin, researchers are developing eco-friendly composites with reduced environmental impact. As our understanding of biological composites and biologically inspired approaches of fabrication of novel biomimetic composites continues to advance, we can expect to see further innovations in fields ranging from healthcare to infrastructure.

In addition to their potential engineering implications, the study of biological composite materials offers valuable insights into the fundamental principles governing biological systems. By applying techniques from materials science, biologists can gain a deeper understanding of the structure–function relationships in organisms and the mechanisms underlying their remarkable properties (see reviews [1–5]). For instance, the hierarchical organization of biological composites, spanning multiple length scales from nanometres to centimetres, provides a blueprint for designing materials with precise control over mechanical, optical and thermal properties. By unraveling the structural motifs and assembly mechanisms found in natural composites, researchers can elucidate the evolutionary adaptations that have enabled organisms to thrive in diverse environments. Moreover, studying biological composite materials allows biologists to appreciate the interplay between form and function in living organisms. From the microarchitecture of bone tissue to the intricate patterns of spider silk, each composite structure is finely tuned to perform specific biological tasks, such as support, protection or locomotion.

Chitin, a polysaccharide polymer found abundantly in the exoskeletons of arthropods, radular teeth of molluscs, cell walls of fungi and certain algae, serves as the primary building block for numerous biological composites. When combined with proteins, minerals or other biopolymers, chitin forms hierarchical structures with unique mechanical, chemical and biological properties (see reviews [6–8]). Recent research has focused on understanding the processes of synthesis, structural organization and functional characteristics of chitin-based composites, elucidating their potential for tissue engineering scaffolds, wound dressings, drug delivery systems, and environmental remediation. Furthermore, chitin's biocompatibility, biodegradability and versatility make it an attractive candidate for sustainable and eco-friendly materials. This theme issue explores the synthesis, properties, and applications of chitin-based biological composite materials, highlighting their importance in addressing current challenges and driving innovation in various technological domains.

In the first contribution [9], insects, with their diverse structures and material properties, are taken as valuable models for studying biological composites. The mouthparts of flower and nonflower visiting moths exhibit distinct morphologies and material properties that correlate with their feeding habits. By employing imaging techniques, such as scanning electron microscopy and confocal laser scanning microscopy, the authors have identified patterns of autofluorescence and surface roughness in moth proboscises, shedding light on their adaptations to feeding on different sources. With regard to the interplay between organisms and food, controlled feeding experiments have been performed in the next contribution with crickets [10]. The investigations of the mandible wear provide insights into diet-dependent microwear patterns. This study introduces and underscores the utility of microwear analysis in inferring invertebrate diets and understanding their feeding behaviours. Also, the paper by Wegst *et al*. [11] shows that due to the interaction with hard and abrasive food, the insect mandibles endure significant loading and wear. In their study, the authors employed element-sensitive X-ray tomography to uncover the presence of zinc in the cutting edges of mandibles, enhancing their stiffness, hardness and wear resistance. They revealed that the locust mandibles enable a scissor-like cutting mechanism and provide a tool, which sharpens itself as the mouthparts shear past one another during feeding.

The mandibles of leafcutter ants showcase structures and mechanical properties tailored to their respective roles within the colony. As analysed by Birkenfeld *et al*. [12], the hardness and Young's modulus of mandibles in the leafcutter ant *Atta laevigata* increase with caste size, with higher concentrations of the transition metal zinc. The regional variations in cuticular composition and mechanical properties have a high impact on the mandible stress patterns, as shown for the ant species *Formica cunicularia* [13]. Finite element analysis on mandible models with homogeneous and heterogeneous material property distribution reveals the impact of these variations on mandible stress patterns under bite loading, highlighting the importance of understanding the regional cuticle material properties.

Similarly to insect mouthparts, the radular teeth of the squid *Loligo vulgaris* have to cope with wear as well. Mechanisms to maintain a high functionality were investigated using various methods, shedding light on the composition and mechanical properties of the teeth [14]. Here, authors revealed that tooth regions prone to wear exhibited greater hardness and stiffness, while surfaces interacting with ingesta displayed a protective coating rich in silicon, likely mitigating abrasion. These findings demonstrate the importance of systematic studies on radular wear for understanding the intricate relationship between tooth structure and properties in molluscan groups.

Besides food processing, chitin-based insect cuticle enables many other kinds of mechanical functions. The locust *Locusta migratoria*, known for its impressive aerial prowess, relies heavily on its hind wings for generating lift and thrust during flight. Zhou *et al.* [15] investigated mechanical properties of cross-veins across different regions of the hind wing using tensile tests, relaxation tests and fluorescence microscopy, revealing these structures to be composed of viscoelastic materials with region-specific differences in elastic moduli and ultimate tensile stress. These findings provide valuable insights into the mechanical behaviour of wings, contributing to a better understanding of the aerodynamics of flapping flight.

From biological structures to bioinspired design, Toofani *et al.* [16] highlight the critical necessity for a novel framework addressing sustainable and regenerative design, proposing ‘complexity biomechanics' as a solution. Complexity biomechanics offers a holistic approach to analyse natural mechanical systems, and to provide a roadmap for developing intelligent engineering materials and structures capable of automatic adaptation to dynamic environments. By applying this framework to dragonfly wings, the study demonstrates how different structural components collaboratively contribute to the functionality of the entire wing system.

The tanned arthropod cuticle provides essential strength, protection, and lightweight properties, yet its limited expandability necessitates periodic moulting for growth. Investigating the temporal changes in sclerotization and pigmentation during and after moulting, the study by Preuss *et al.* [17] on the tarsal and mandibular cuticle of the dragonfly *Anax imperator* reveals differing tanning rates and patterns. Specifically, the faster tanning of the tarsal cuticle compared to the mandibular cuticle suggests a demand for increased mechanical stability in structures involved in emergence and initial locomotion or hunting attempts.

Recent research on crustacean cuticles has uncovered diverse fibre architectures and mineral compositions, revealing adaptations to various mechanical demands beyond the standard twisted plywood model. As provided in a comprehensive review by Vittori [18], these cuticles exhibit intricate patterns, such as longitudinal and circular parallel fibre arrays with skeletal minerals including calcium phosphates and calcium carbonates. Moreover, protein cross-linking influences skeletal properties, serving as a stiffening mechanism. These concepts offer biomimetic ideas for future technological developments.

As emphasized by this theme issue, the cooperative approach of biology and materials science in the study of biological composite materials not only fosters technological innovation but also deepens our understanding of the organisms evolved in the course of biological evolution. By bridging these disciplines, researchers can advance both our knowledge of biology and our ability to engineer materials with unprecedented properties and functionalities.

## Data Availability

This article has no additional data.
